# Color center creation by dipole stacking in crystals of 2-meth­oxy-5-nitro­aniline

**DOI:** 10.1107/S2056989024008739

**Published:** 2024-09-10

**Authors:** Jonathan Filley

**Affiliations:** a Oligometrics, Inc., 2510 47th Street, Suite 208, Boulder, CO, 80301, USA; University of Missouri-Columbia, USA

**Keywords:** crystal structure, color center, dipole stacking

## Abstract

This work describes the X-ray structure of orange–red crystals of 2-meth­oxy-5-nitro­aniline, C_7_H_8_N_2_O_3_. The compound displays concentration-dependent UV-Vis spectra, which is attributed to dipole-induced aggregation, and light absorption arising from an inter­molecular charge-transfer process that decreases in energy as the degree of aggregation increases. The crystals display π-stacking where the dipole moments align anti­parallel. Stacked mol­ecules inter­act with the next stack *via* hydrogen bonds, which is a state of maximum aggregation. Light absorption by charge transfer can be compared to colored inorganic semiconductors such as orange–red CdS, with a band gap of 2.0–2.5 eV.

## Abstract 2

The title compound 2-meth­oxy-5-nitro­aniline forms orange–red crystals and displays shifts in light absorption to longer wavelengths in solution as the concentration increases. Mol­ecular face-to-face stacking with dipoles oriented anti­parallel creates a color center, which is compared to certain inorganic semiconductors.

## 1. Chemical context

**Figure 1:**
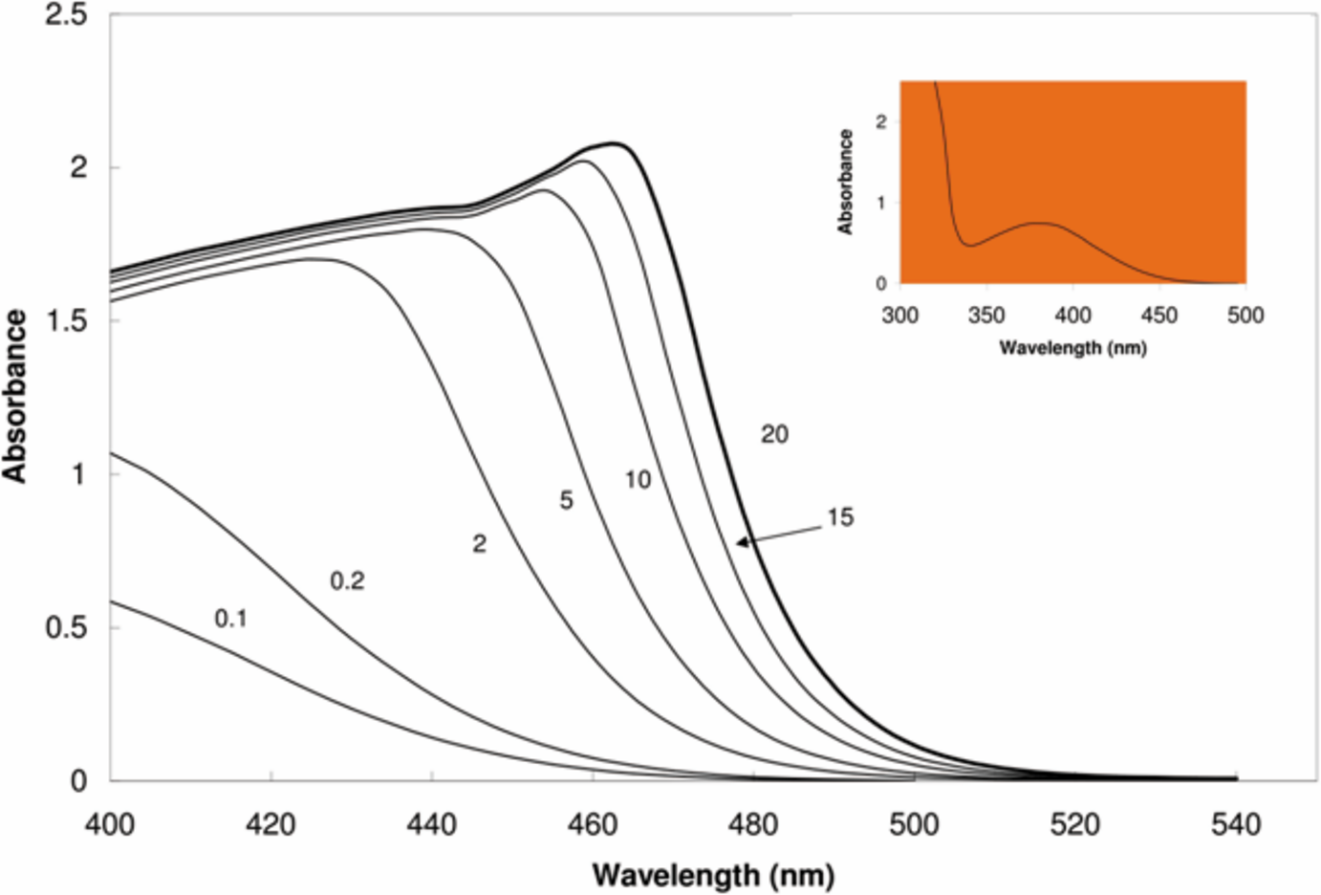
Concentration-dependent spectra in acetone in the visible region for 2-meth­oxy-5-nitro­aniline (concentrations in m*M*). Inset: UV-Vis spectrum (0.1 m*M*).

The title compound is an inexpensive and versatile starting material with two chemically distinct nitro­gen atoms that can be functionalized is a variety of ways. The mol­ecule features *para*-oriented electron-donating and withdrawing groups as shown in the scheme, giving rise to a large dipole moment of 5.4 D (Buemi *et al.* 1979 ▸). During routine crystallization prior to its use in synthesis, we were struck by the beauty of its orange–red crystals and the ease of their formation. While dilute solutions of the compound in acetone are yellow (λ_max_ = 380 nm), more concentrated solutions exhibit a striking longer wavelength absorption, which moves further into the visible portion of the spectrum as the concentration increases (Fig. 1 ▸). These spectra are consistent with a concentration-dependent aggregation phenomenon aided by the strong dipole moment, which results in inter­molecular charge transfer, where the electronic transition comes at lower energies as the degree of aggregation increases. Presumably, at higher concentrations, light absorption would approach 490 nm, which would result in an orange–red solution according to chromaticity diagrams (Nassau, 1983 ▸). These concentrations cannot be achieved due to solubility limitations, but solid material with no solvent mol­ecules can be considered a state of maximum aggregation.
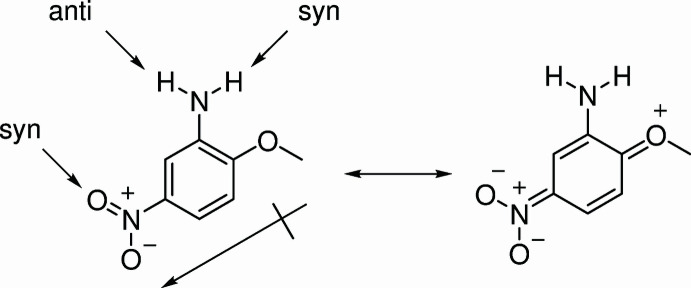


Crystals of substituted anilines comparable to 2-meth­oxy-5-nitro­aniline have inter­esting non-linear optical properties (Ravikumara & Hubert Joe, 2010 ▸) and have been the subject of structure investigations (Rosli *et al.*, 2007 ▸). We undertook an X-ray structure study of the title compound to help understand the color of the crystals, and found a π-stacked face-to-face arrangement of the mol­ecules with dipoles aligned anti­parallel, which can facilitate a charge-transfer mechanism for light absorption. The color of the title compound is akin to that seen in certain inorganic semiconductors such as CdS, where a band gap of 2.0–2.5 eV gives a similar orange–red color, and is the result of charge transfer within the crystal from the valence band to the conduction band (Pal *et al.*, 1997 ▸).

## 2. Structural commentary

**Figure 2:**
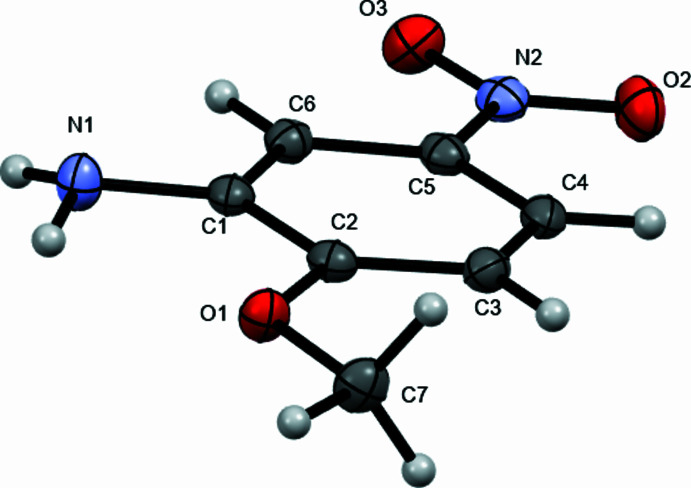
Mol­ecular structure of 2-meth­oxy-5-nitro­aniline showing the pyramidal amino group. Atoms are displayed as ellipsoids at the 50% probability displacement level.

The mol­ecular structure in Fig. 2 ▸ shows the pyramidal amino group, suggesting the amino group lone-pair electrons are not highly conjugated with the aromatic ring, consistent with an amino group *meta* to a nitro group.

## 3. Supra­molecular features

**Figure 3:**
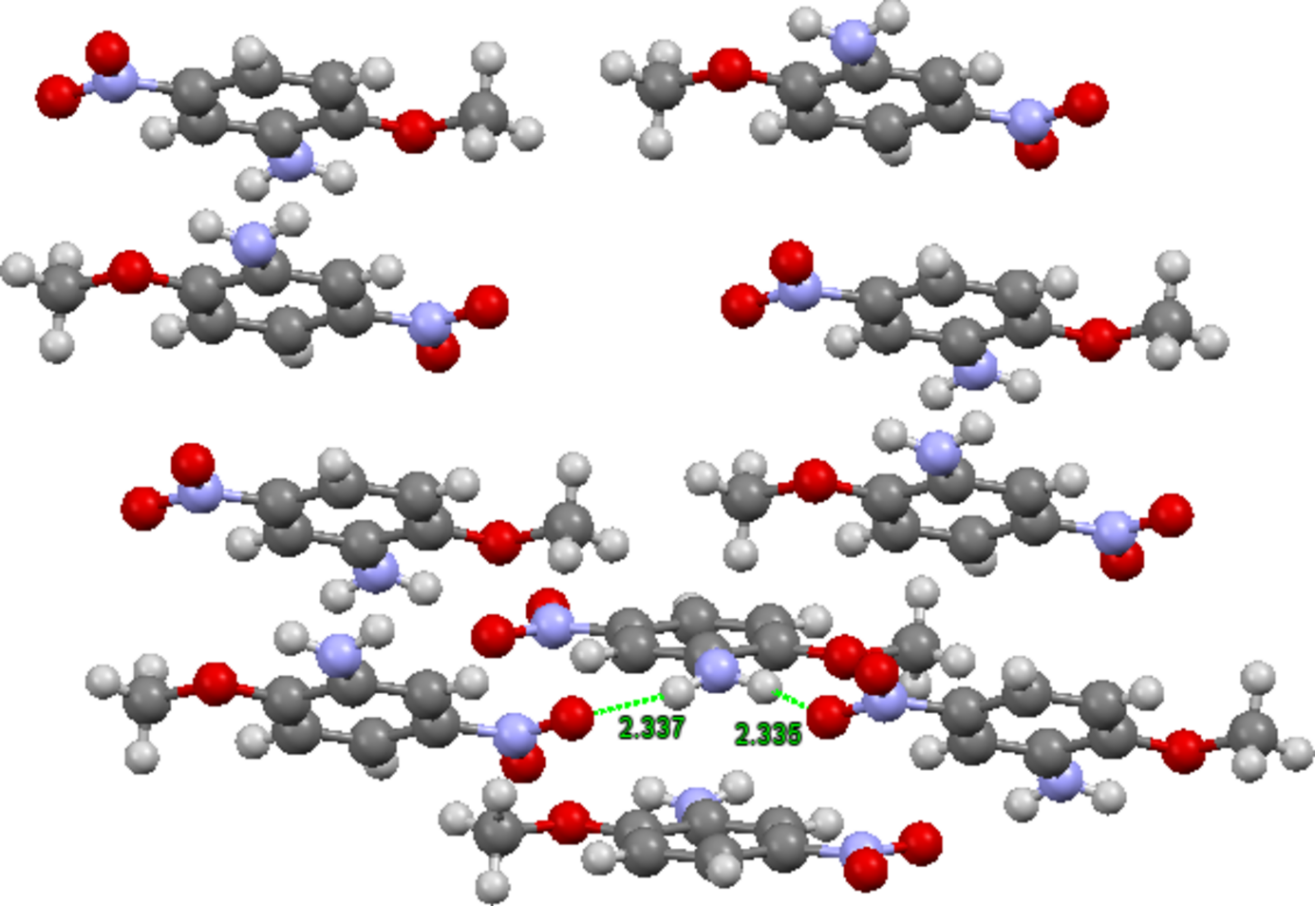
Stacked mol­ecules of the title compound showing anti­parallel aligned dipoles and hydrogen bonding from the amino hydrogen atoms to *syn* nitro oxygen atoms.

Key features of the crystal packing are displayed in Fig. 3 ▸. It can be seen that on a pairwise basis, the mol­ecules stack on top of each other with almost perfect alignment of the meth­oxy group of one mol­ecule and the nitro group of the next mol­ecule, with the dipoles oriented anti­parallel. Indeed, this is generally observed in crystals of mol­ecules with non-zero dipole moments, and in fact it is an ongoing challenge to design crystals with parallel dipole moments, since these are expected to have strong non-linear optical properties (Lewis *et al.*, 2000 ▸). Fig. 3 ▸ also shows an offset of about 1.7 Å for the next pair of stacked mol­ecules. The stacked columns are connected by hydrogen bonds with lengths of 2.34 (2) Å (Table 1 ▸). Inter­estingly, these inter-stack hydrogen bonds connect both *syn* and *anti* amino group hydrogen atoms on one stack to only the *syn* oxygen atoms of the nitro group on a different stack (the *anti* nitro oxygen atoms are not involved in hydrogen bonds). In order for this to be true, the stacks that bear nitro group oxygen atom hydrogen-bond acceptors have to be parallel, and the *syn* acceptors are flipped with respect to each other. The angle between the planes defining the two stacks is 33.6  (7)°. It is hypothesized that the observed orange–red color of the crystals arises from inter­molecular charge transfer, amounting to a color center and colored semiconductor-like behavior.

## 4. Database survey

Related nitro anilines such as 4-meth­oxy-2-nitro­aniline have been subjected to X-ray structure analysis (Rosli *et al.*, 2007 ▸), which shows a near planar amino group (the amino and nitro groups are *ortho* to each other and are therefore conjugated) and a slightly shorter N—H⋯O hydrogen bond of 2.20 Å. A neutron diffraction study found a slightly pyramidal amino group in 2-methyl-4-nitro­aniline (Whitten *et al.*, 2006 ▸). The compound 2-bromo-4-nitro­aniline has inter­molecular hydrogen bonding almost identical to that reported here (Arshad *et al.*, 2009 ▸).

## 5. Synthesis and crystallization

2-Meth­oxy-5-nitro­aniline was obtained from Aldrich and recrystallized from methanol. UV-VIS spectra were collected on a Perkin–Elmer Lambda 3A spectrophotometer. Spectra were collected using a path length of 1.0 cm in acetone solution. Images of mol­ecular structures were manipulated using *Mercury* (Macrae *et al.*, 2020 ▸).

## 6. Refinement

Crystal data, data collection and structure refinement details are summarized in Table 2 ▸.

## Supplementary Material

Crystal structure: contains datablock(s) I. DOI: 10.1107/S2056989024008739/ev2011sup1.cif

Structure factors: contains datablock(s) I. DOI: 10.1107/S2056989024008739/ev2011Isup2.hkl

CCDC reference: 2382128

Additional supporting information:  crystallographic information; 3D view; checkCIF report

## Figures and Tables

**Table 1:** Hydrogen-bond geometry (Å, °)

*D*—H⋯*A*	*D*—H	H⋯*A*	*D*⋯*A*	*D*—H⋯*A*
C4—H4⋯O1^i^	0.952 (16)	3.177 (15)	3.7473 (14)	120.2 (11)
C3—H3⋯O1^i^	0.944 (15)	3.039 (15)	3.6820 (14)	126.7 (11)
C3—H3⋯O2^ii^	0.944 (15)	2.646 (15)	3.3523 (14)	132.1 (11)
C6—H6⋯O1^iii^	0.958 (16)	3.006 (16)	3.7075 (14)	131.2 (11)
C6—H6⋯O2^iv^	0.958 (16)	2.527 (15)	3.3090 (14)	138.8 (12)
C6—H6⋯N2^iv^	0.958 (16)	3.130 (16)	3.9611 (14)	146.0 (12)
C7—H7*A*⋯O2^ii^	1.001 (17)	2.691 (17)	3.6087 (16)	152.5 (12)
C7—H7*B*⋯O3^v^	0.986 (16)	3.130 (15)	3.9801 (16)	145.2 (11)
C7—H7*B*⋯N1^i^	0.986 (16)	2.990 (16)	3.8090 (16)	141.1 (11)
N1—H1*A*⋯O3^vi^	0.885 (17)	2.335 (17)	3.0395 (13)	136.6 (14)
C7—H7*C*⋯N1^vi^	0.962 (16)	2.847 (16)	3.6953 (17)	147.6 (12)
N1—H1*B*⋯O3^iv^	0.869 (19)	2.337 (19)	3.1812 (13)	163.9 (15)
N1—H1*B*⋯O2^iv^	0.869 (19)	2.903 (18)	3.6404 (14)	143.7 (14)
N1—H1*B*⋯N2^iv^	0.869 (19)	2.944 (19)	3.7781 (14)	161.3 (14)

Symmetry codes: (i) 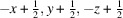; (ii) 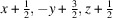; (iii) 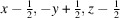; (iv) 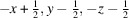; (v) 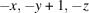; (vi) 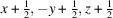.

**Table 2:** Experimental details

Crystal data	
Chemical formula	C_7_H_8_N_2_O_3_
*M*_r_	168.15
Crystal system, space group	Monoclinic, *P*2_1_/*n*
Temperature (K)	100
*a*, *b*, *c* (Å)	7.14981 (11), 9.79512 (11), 10.74206 (14)
β (°)	96.9437 (14)
*V* (Å^3^)	746.78 (2)
*Z*	4
Radiation type	Cu *K*α
μ (mm^−1^)	1.01
Crystal size (mm)	0.5 × 0.4 × 0.2

Data collection	
Diffractometer	Xcalibur, Onyx, Ultra
Absorption correction	Multi-scan (*CrysAlis PRO*; Agilent, 2014 ▸)
*T*_min_, *T*_max_	0.827, 1.000
No. of measured, independent and observed [*I* > 2σ(*I*)] reflections	13822, 1543, 1398
*R*_int_	0.049
(sin θ/λ)_max_ (Å^−1^)	0.630

Refinement	
*R*[*F*^2^ > 2σ(*F*^2^)], *wR*(*F*^2^), *S*	0.035, 0.097, 1.06
No. of reflections	1543
No. of parameters	141
H-atom treatment	All H-atom parameters refined
Δρ_max_, Δρ_min_ (e Å^−3^)	0.25, −0.17

Computer programs: *CrysAlis PRO* (Agilent, 2014 ▸), *SHELXS* (Sheldrick, 2008 ▸), *SHELXL2013/4* (Sheldrick, 2015 ▸) and *OLEX2* (Dolomanov *et al.*, 2009 ▸).

## supplementary crystallographic information

### 2-Methoxy-5-nitroaniline Crystal data

**Table:**

C_7_H_8_N_2_O_3_	*F*(000) = 352
*M**_r_* = 168.15	*D*_x_ = 1.496 Mg m^−^^3^
Monoclinic, *P*2_1_/*n*	Cu *K*α radiation, λ = 1.54184 Å
*a* = 7.14981 (11) Å	Cell parameters from 7580 reflections
*b* = 9.79512 (11) Å	θ = 4.1–75.9°
*c* = 10.74206 (14) Å	µ = 1.01 mm^−^^1^
β = 96.9437 (14)°	*T* = 100 K
*V* = 746.78 (2) Å^3^	Block, clear dark orange
*Z* = 4	0.5 × 0.4 × 0.2 mm

### 2-Methoxy-5-nitroaniline Data collection

**Table:**

Xcalibur, Onyx, Ultra diffractometer	1543 independent reflections
Radiation source: sealed X-ray tube, Enhance Ultra (Cu) X-ray Source	1398 reflections with *I* > 2σ(*I*)
Mirror monochromator	*R*_int_ = 0.049
Detector resolution: 8.2603 pixels mm^-1^	θ_max_ = 76.1°, θ_min_ = 7.1°
ω scans	*h* = −8→8
Absorption correction: multi-scan (CrysAlisPro; Agilent, 2014)	*k* = −12→12
*T*_min_ = 0.827, *T*_max_ = 1.000	*l* = −13→13
13822 measured reflections	

### 2-Methoxy-5-nitroaniline Refinement

**Table:**

Refinement on *F*^2^	Primary atom site location: structure-invariant direct methods
Least-squares matrix: full	Hydrogen site location: difference Fourier map
*R*[*F*^2^ > 2σ(*F*^2^)] = 0.035	All H-atom parameters refined
*wR*(*F*^2^) = 0.097	*w* = 1/[σ^2^(*F*_o_^2^) + (0.0542*P*)^2^ + 0.2402*P*] where *P* = (*F*_o_^2^ + 2*F*_c_^2^)/3
*S* = 1.06	(Δ/σ)_max_ < 0.001
1543 reflections	Δρ_max_ = 0.25 e Å^−^^3^
141 parameters	Δρ_min_ = −0.16 e Å^−^^3^
0 restraints	

### 2-Methoxy-5-nitroaniline Special details

**Table:**

Geometry. All esds (except the esd in the dihedral angle between two l.s. planes) are estimated using the full covariance matrix. The cell esds are taken into account individually in the estimation of esds in distances, angles and torsion angles; correlations between esds in cell parameters are only used when they are defined by crystal symmetry. An approximate (isotropic) treatment of cell esds is used for estimating esds involving l.s. planes.

### 2-Methoxy-5-nitroaniline Fractional atomic coordinates and isotropic or equivalent isotropic displacement parameters (Å^2^)

**Table:**

	*x*	*y*	*z*	*U*_iso_*/*U*_eq_	
O1	0.43312 (12)	0.35947 (8)	0.24323 (7)	0.0204 (2)	
O3	0.09878 (13)	0.51541 (9)	−0.30006 (8)	0.0259 (2)	
O2	0.05943 (13)	0.70793 (9)	−0.20810 (9)	0.0278 (2)	
C3	0.28683 (16)	0.55666 (12)	0.13260 (11)	0.0192 (3)	
N2	0.11198 (14)	0.58793 (10)	−0.20487 (9)	0.0201 (2)	
N1	0.38353 (15)	0.20274 (10)	0.04027 (10)	0.0212 (2)	
C5	0.19259 (16)	0.53005 (11)	−0.08607 (10)	0.0178 (3)	
C1	0.32888 (16)	0.33783 (11)	0.02968 (11)	0.0172 (3)	
C6	0.24983 (16)	0.39389 (12)	−0.08308 (10)	0.0177 (3)	
C2	0.34803 (16)	0.42203 (12)	0.13800 (10)	0.0174 (3)	
C4	0.20887 (16)	0.61213 (12)	0.01915 (11)	0.0190 (3)	
C7	0.45834 (19)	0.43980 (13)	0.35597 (11)	0.0231 (3)	
H4	0.167 (2)	0.7045 (16)	0.0136 (14)	0.025 (4)*	
H3	0.301 (2)	0.6112 (15)	0.2055 (14)	0.022 (3)*	
H6	0.236 (2)	0.3386 (16)	−0.1572 (15)	0.024 (4)*	
H7A	0.532 (2)	0.5240 (17)	0.3417 (14)	0.032 (4)*	
H7B	0.335 (2)	0.4632 (16)	0.3830 (13)	0.027 (4)*	
H1A	0.459 (2)	0.1820 (16)	0.1091 (16)	0.027 (4)*	
H7C	0.527 (2)	0.3821 (16)	0.4180 (14)	0.027 (4)*	
H1B	0.407 (2)	0.1628 (18)	−0.0284 (17)	0.035 (4)*	

### 2-Methoxy-5-nitroaniline Atomic displacement parameters (Å^2^)

**Table:**

	*U*^11^	*U*^22^	*U*^33^	*U*^12^	*U*^13^	*U*^23^
O1	0.0242 (4)	0.0200 (4)	0.0163 (4)	0.0008 (3)	0.0000 (3)	−0.0008 (3)
O3	0.0314 (5)	0.0261 (5)	0.0197 (4)	−0.0031 (4)	0.0004 (3)	0.0019 (3)
O2	0.0309 (5)	0.0211 (4)	0.0317 (5)	0.0056 (4)	0.0046 (4)	0.0078 (4)
C3	0.0174 (6)	0.0190 (6)	0.0215 (6)	−0.0021 (4)	0.0036 (4)	−0.0039 (4)
N2	0.0175 (5)	0.0207 (5)	0.0225 (5)	−0.0020 (4)	0.0037 (4)	0.0045 (4)
N1	0.0264 (6)	0.0183 (5)	0.0185 (5)	0.0029 (4)	0.0012 (4)	−0.0010 (4)
C5	0.0142 (5)	0.0196 (6)	0.0199 (6)	−0.0012 (4)	0.0034 (4)	0.0029 (4)
C1	0.0144 (5)	0.0174 (5)	0.0202 (5)	−0.0017 (4)	0.0042 (4)	−0.0003 (4)
C6	0.0165 (6)	0.0191 (5)	0.0180 (5)	−0.0016 (4)	0.0040 (4)	−0.0015 (4)
C2	0.0146 (6)	0.0199 (6)	0.0179 (5)	−0.0014 (4)	0.0025 (4)	0.0013 (4)
C4	0.0149 (6)	0.0165 (5)	0.0263 (6)	0.0001 (4)	0.0050 (4)	0.0006 (4)
C7	0.0265 (7)	0.0249 (6)	0.0176 (6)	−0.0007 (5)	0.0010 (5)	−0.0023 (5)

### 2-Methoxy-5-nitroaniline Geometric parameters (Å, º)

**Table:**

O1—C2	1.3627 (14)	N1—H1B	0.869 (19)
O1—C7	1.4373 (14)	C5—C6	1.3943 (16)
O3—N2	1.2393 (13)	C5—C4	1.3804 (16)
O2—N2	1.2332 (13)	C1—C6	1.3866 (16)
C3—C2	1.3884 (16)	C1—C2	1.4193 (16)
C3—C4	1.3881 (16)	C6—H6	0.958 (16)
C3—H3	0.944 (15)	C4—H4	0.952 (16)
N2—C5	1.4506 (14)	C7—H7A	1.001 (17)
N1—C1	1.3805 (15)	C7—H7B	0.986 (16)
N1—H1A	0.885 (17)	C7—H7C	0.962 (16)
			
C2—O1—C7	116.79 (9)	C5—C6—H6	121.4 (9)
C2—C3—H3	120.1 (9)	C1—C6—C5	119.10 (10)
C4—C3—C2	119.97 (10)	C1—C6—H6	119.5 (9)
C4—C3—H3	119.9 (9)	O1—C2—C3	124.65 (10)
O3—N2—C5	118.97 (9)	O1—C2—C1	114.07 (10)
O2—N2—O3	122.04 (10)	C3—C2—C1	121.26 (10)
O2—N2—C5	118.99 (10)	C3—C4—H4	121.1 (9)
C1—N1—H1A	115.3 (10)	C5—C4—C3	118.43 (10)
C1—N1—H1B	116.3 (11)	C5—C4—H4	120.4 (9)
H1A—N1—H1B	116.4 (15)	O1—C7—H7A	109.7 (9)
C6—C5—N2	118.58 (10)	O1—C7—H7B	110.4 (9)
C4—C5—N2	118.54 (10)	O1—C7—H7C	105.3 (9)
C4—C5—C6	122.87 (11)	H7A—C7—H7B	111.0 (13)
N1—C1—C6	122.20 (10)	H7A—C7—H7C	110.9 (13)
N1—C1—C2	119.42 (10)	H7B—C7—H7C	109.3 (12)
C6—C1—C2	118.35 (10)		
			
O3—N2—C5—C6	−0.95 (16)	C6—C1—C2—O1	−177.80 (9)
O3—N2—C5—C4	179.20 (10)	C6—C1—C2—C3	1.01 (17)
O2—N2—C5—C6	179.34 (10)	C2—C3—C4—C5	0.67 (17)
O2—N2—C5—C4	−0.51 (16)	C2—C1—C6—C5	0.09 (17)
N2—C5—C6—C1	179.34 (9)	C4—C3—C2—O1	177.28 (10)
N2—C5—C4—C3	−179.72 (10)	C4—C3—C2—C1	−1.41 (17)
N1—C1—C6—C5	178.05 (10)	C4—C5—C6—C1	−0.82 (18)
N1—C1—C2—O1	4.18 (15)	C7—O1—C2—C3	0.66 (16)
N1—C1—C2—C3	−177.01 (10)	C7—O1—C2—C1	179.43 (9)
C6—C5—C4—C3	0.44 (18)		

### 2-Methoxy-5-nitroaniline Hydrogen-bond geometry (Å, º)

**Table:**

*D*—H···*A*	*D*—H	H···*A*	*D*···*A*	*D*—H···*A*
C4—H4···O1^i^	0.952 (16)	3.177 (15)	3.7473 (14)	120.2 (11)
C3—H3···O1^i^	0.944 (15)	3.039 (15)	3.6820 (14)	126.7 (11)
C3—H3···O2^ii^	0.944 (15)	2.646 (15)	3.3523 (14)	132.1 (11)
C6—H6···O1^iii^	0.958 (16)	3.006 (16)	3.7075 (14)	131.2 (11)
C6—H6···O2^iv^	0.958 (16)	2.527 (15)	3.3090 (14)	138.8 (12)
C6—H6···N2^iv^	0.958 (16)	3.130 (16)	3.9611 (14)	146.0 (12)
C7—H7*A*···O2^ii^	1.001 (17)	2.691 (17)	3.6087 (16)	152.5 (12)
C7—H7*B*···O3^v^	0.986 (16)	3.130 (15)	3.9801 (16)	145.2 (11)
C7—H7*B*···N1^i^	0.986 (16)	2.990 (16)	3.8090 (16)	141.1 (11)
N1—H1*A*···O3^vi^	0.885 (17)	2.335 (17)	3.0395 (13)	136.6 (14)
C7—H7*C*···N1^vi^	0.962 (16)	2.847 (16)	3.6953 (17)	147.6 (12)
N1—H1*B*···O3^iv^	0.869 (19)	2.337 (19)	3.1812 (13)	163.9 (15)
N1—H1*B*···O2^iv^	0.869 (19)	2.903 (18)	3.6404 (14)	143.7 (14)
N1—H1*B*···N2^iv^	0.869 (19)	2.944 (19)	3.7781 (14)	161.3 (14)

Symmetry codes: (i) −*x*+1/2, *y*+1/2, −*z*+1/2; (ii) *x*+1/2, −*y*+3/2, *z*+1/2; (iii) *x*−1/2, −*y*+1/2, *z*−1/2; (iv) −*x*+1/2, *y*−1/2, −*z*−1/2; (v) −*x*, −*y*+1, −*z*; (vi) *x*+1/2, −*y*+1/2, *z*+1/2.
